# The Keele community knee pain forum: action research to engage with stakeholders about the prevention of knee pain and disability

**DOI:** 10.1186/1471-2474-10-85

**Published:** 2009-07-15

**Authors:** Clare Jinks, Bie Nio Ong, Tracey J O'Neill

**Affiliations:** 1 Arthritis Research Campaign National Primary Care Centre, Keele University Keele, UK

## Abstract

**Background:**

Involvement of users in health care research is central to UK health care policy, and guidelines for involvement exist. However, there are limited examples in rheumatology research. The aim of this study was to establish a community knee pain forum aimed at engaging stakeholders in design, dissemination and prioritisation of knee pain research.

**Methods:**

Ten people were recruited to the forum representing a wide range of agencies. These included Weight Watchers, the leisure industry, Beth Johnson Foundation, health and social care professionals and the public. Three two-hour meetings over a two-year period were held. Experienced qualitative researchers facilitated each meeting. Written feedback after each meeting was elicited, and a short evaluation form was mailed to all members after the final meeting.

**Results:**

Establishing and maintaining a forum of mixed members required careful preparation and ongoing support. Meetings had to be well-structured in order to allow for balanced participation of lay and professional users. Users contributed to the design of methods, provided ideas for dissemination and set priorities for further research. Clear documentation of meetings ensured that users' contributions to the research cycle were transparent.

**Conclusion:**

Our knee pain forum illustrates that community engagement can have a positive impact on the development, dissemination and implementation of health research. Engaging with non-academic partners enables mutual learning and this enhances the quality of NHS research.

## Background

The need to involve users in health care research is now explicit in UK health care policy [1] and a model for involvement in research and development has been developed by INVOLVE. This model [2] comprises 3 levels of involvement (consultation, collaboration and user control), and includes nine stages, for example, identifying and prioritising topics for research, designing, managing, commissioning, undertaking and disseminating research.

INVOLVE define the need for "an active partnership between consumers and researchers in the research process, rather than the use of consumers as the subjects of research"[3]. Another approach is "user controlled research" which is research that users initiate and develop themselves rather than research which has been initiated by others [4].

Many benefits of user involvement in research have been reported. Boote et al [5] argue that "consumers' experiential knowledge can add synergy to the traditional disease focus of health research, and can facilitate the generation of more relevant research questions [6] and outcome measures." Patients often have insights and expertise that complement those of health care professionals and their involvement in research may thus improve quality and impact, add legitimacy and value, identify research gaps and improve uptake of research findings [6-10]. Finally, there exists a moral argument that, "as citizens and "owners" of the NHS, consumers are entitled to have a voice about research issues in their health service" [11].

When planning, undertaking and disseminating health research, numerous challenges exist for researchers, non-academic partners and research funders. Firstly, there is a lack of strategies to facilitate structural participation in the research process [12] including sustainable ways to integrate experiential and scientific knowledge. Secondly, researchers may be opposed to the concept of user participation and feel that user views are insignificant and carry little weight in the research process [11,13]. It has been argued that patients lack objective knowledge and that this inhibits a substantive contribution to be made [12]. A common criticism of user involvement is that it suffers from a lack of representativeness of those involved. However, as Beresford notes other stakeholders are not expected to be representative and the issue is thus one of seeking diversity and inclusivity [4]. A key challenge to user involvement is that of communication, and problems emerge as patients, professionals, researchers and clinicians do not share a common language. The most important issue is "not whether a patient will say something but whether the patient gains a hearing and is taken seriously"[7]. Tokenism can occur where patients share insights but are not given recognition for their contribution and have minimal influence over the research process.

Criteria for successful consumer involvement in NHS research have been published [2,14]. These include principles such as agreed roles, appropriate budgets, respect, training and support, appropriate skills of researchers, joint decision making, and acknowledgement and dissemination of user input. Despite recent criteria, and a general increase in research involving users in the UK, there are few examples of user involvement in rheumatology research and most work has been in the field of rheumatoid arthritis (RA) [15-19]. Wider community engagement in musculoskeletal research is thus limited, and the reasons for that are not wholly clear.

We gained funding for a Knee Pain Prevention Project (KNEPP) and wanted to involve users in various stages of the research process, namely designing methods, disseminating findings and prioritising future research. Historically research methods have been defined by researchers and user participation is still low due to the continuing use of established research designs [7]. The importance of involving patients in research design is that they can think clearly about the practicalities of carrying out research and the outcome measures that are used [3].

The KNEPP study had three linked parts:

1) A systematic review of risk factors for onset and progression of knee pain in the community, including modelling of risk factors with existing survey data to identify the effect of reducing risk factors in the population.

2) A NHS record review to assess inequalities in access to health care for knee pain in older adults. General practice and hospital records were searched.

3) A qualitative study to identify perceptions of knee pain prevention in adults aged 50 years and over, and in people who work with older people or people with knee pain.

We established a community knee pain forum as the place where design and methods, dissemination and prioritisation of topics could be explored. We wanted a setting in which patients and the public, voluntary and community organisations and social and health care agencies could work in partnership.

We were influenced by the desire to include a wide range of perspectives in the research process. Hence our definition of users was broad. We defined users as patients with knee pain, but also as people who we hope will ultimately "use" the results of the research for patient benefit (e.g. local health or community group workers). This approach is somewhat different in that a mixed group was convened. We felt that an inclusive approach was required in order to get to grips with the many aspects of knee pain prevention that the project aimed to cover.

This paper reports on how user involvement was defined and operationalised within the knee pain forum, and offers a preliminary assessment of its outcomes. The project received full ethical approval from South Staffordshire Research Ethics Committee (Ref 05/Q2602/37).

## Methods

Key aspects of INVOLVE's model of user involvement were used to define the specific aims of the knee pain forum. In particular we wanted help and advice from the users on issues of disseminating the results of part one of the KNEPP study (the systematic review on risk factors). We were also committed to involving users in the design of the methods for part three of the KNEPP Study (the qualitative study). We wanted to undertake qualitative interviews with people about their understanding of the prevention of knee pain and disability in older adults and what the key determinants of this were. Identifying determinants of health is a complex topic and thus we wanted to discuss possible interview methods and questions with the forum. Finally we wanted to explore what people thought should be priorities for future research.

In setting up the group, the first task was to create the context within which the group could operate. This preparatory phase is about identifying stakeholders, informing them of the study and gaining consent to participate. As Abma notes, "the social conditions for dialogue are not given but should be actively created [7]."

We adopted a snowballing technique to identify potential forum members, initially by using our local knowledge of health services, voluntary and community organisations. So, for example, we first targeted key organisations (like weight watchers) and then asked them to suggest other groups to invite. We wrote to people explaining the study, and the role that the forum had within the study. In this way we recruited 10 people from a wide range of agencies (see table 1). Half of these members were "traditional" users. By this we mean that they were non-academic or non-NHS representatives. In addition were 3 university researchers, bringing the total number to 13 members overall. There were no expectations that different stakeholders had different roles in the forum. We aimed to bring together a mixed group who shared a common interest in using the results of the research.

**Table 1:** Profile of knee pain forum members

**Stakeholder**	**Gender**	**Number of meetings attended**
Arthritis Care & Lay member	Female	3
Arthritis Care & Lay member	Male	3
Beth Johnson Foundation (older person's role)	Female	1
General Practitioner	Male	3
Health Promotion (general)	Female	3
Health Promotion (activity and older people)	Female	2
Leisure Centre (Fitness Instructor)	Male	1
Community Physiotherapist	Female	3
Researcher	Female	3
Researcher	Female	3
Researcher	Female	1
Social Services (Older people)	Female	1
Weight watchers coordinator	Female	2

We followed good practice guidelines to promote participation in the meetings and carefully prepared all meetings [20]. This included sending materials in advance, inclusive seating arrangements, small and whole group work. Each meeting had clear objectives and at the end these were revisited and the main conclusions were agreed upon. All three meetings were facilitated by CJ and TO who had previous experience of qualitative interviewing and running focus groups.

The team was cognisant of the need to ensure that lay members did not feel overwhelmed by professionals, as this is a possibility in a mixed group. Support was provided during and in between meetings (e.g. giving extra information) so that lay members could fully contribute. Offering resources to support user involvement is one method of ensuring inclusion in health research [4]. Members of the forum were paid a standard rate of £75 for attending, and paid for travelling and out-of-pocket expenses.

All meetings were held at Keele University, as potential members stated this was a suitable venue. Each meeting was two hours in length. We had three meetings over the two years of the KNEPP study, at key time points in the study. So, for example, the first meeting was held prior to the qualitative study starting so that advice could be gained on potential methods for that study. Feedback on aspects of design implemented as a result of the discussion was then given at the second meeting. Attendance at the meetings varied. It proved difficult to find dates when all members were available. 7 people attended all 3 meetings. 12 people attended the first meeting, 10 the second meeting and 7 people the third meeting.

A short evaluation form was mailed to all members after each meeting, asking for feedback on the key issues discussed, and the process followed. The final evaluation form contained open questions and asked about expectations and experiences of involvement, and about what should be done differently in future. Furthermore, at the last meeting summary conclusions from the forum were debated focusing on key findings, dissemination, and questions for further research. Detailed notes were taken at all meetings, backed up by tape-recording. The themes for analysis were separated between processes at the forum and outcomes that affected the KNEPP study as a whole.

## Results and discussion

Creating the social conditions for the forum was challenging, because of the heterogeneous nature of the group. Individuals came with varying expectations and perspectives. Maintaining the forum was also a challenge, in particular, securing continuous attendance from statutory sector members. We had hoped for a Primary Care Trust (PCT) representative, but this was not forthcoming, and initial resistance from Social Services to engage with the forum hindered recruitment for some time. Colleagues from the Local Strategic Partnership were invited but were not able to participate. We contacted potential members before meetings to ask whether they were attending, with mixed success. The structural and political pressures on statutory organisations may inhibit their engagement, and this needs further exploration.

The economics of user involvement requires consideration. In our study, participants were offered a standard rate of £75 per session. To date our forum has cost £3349.36. These costs need to be included in research proposals. Paying users to be involved in research can be problematic however, as it can affect benefits.

A summary of the key tasks undertaken and the skills required of the researchers is provided in Table 2. Thirteen people participated in the knee pain forum meetings (including three researchers) (see Table 1). Not all members attended all three meetings (range 12 – 7).

**Table 2:** Creating social conditions for a community forum

**Task**	**Key characteristics required**
1. Identify a comprehensive list of potential members from a wide range of agencies. Use local knowledge, colleagues, internet search, snowballing through previous research participants	Patience and persistence to identify people and their correct names and addresses
2. Create a database of names and addresses of potential members	Knowledge of computers
3. Write to potential members explaining the study	Jargon free letter and information sheet
4. Follow up phone call to arrange meeting	Persistence, patience, friendly and approachable nature, enthusiasm
5. Attend a first contact meeting in participants place of work/home to introduce yourself and the project.	Friendly and approachable nature, enthusiasm
6. Write to potential members to establish the best time and date for the meeting. Offer a choice for participants well in advance. Enclose slip and return envelope for people to respond. Request items for the agenda	Organisational and communications skills, persistence and patience
7. Confirm the most convenient date for the meeting with all members (by email, phone, letter)	Organisational and communication skills
8. Mail documentation (agenda and related papers) to members well in advance of the meeting	Organisational and communication skills
9. Maintain contact prior to the meeting	Communication skills
10. Preparation for first meeting: book venue, order refreshments, prepare visual aids, plan group work and activities, prepare handouts, arrange transport.	Organisational and communication skills
11. Meeting Day. Build up a rapport between members and keep presentations to a minimum. Encourage participation from all members. Discuss expectations (and set up evaluation if required).	Friendly and approachable nature, enthusiasm, organisational, communication and facilitation skills. Knowledge of evaluation techniques.
12. Write to members thanking them for their involvement. Include notes of the meeting and agreed action points. Request topics for the next agenda.	Communication skills
13. Commence organisation of next meeting e.g. return to point 6.	Organisational and communications skills, persistence and patience
14. Evaluate participation and outcomes (if required)	Knowledge of evaluation techniques.

The work of the forum in research design, dissemination and identifying future research priorities is now outlined.

### 1. Involvement in research design

**Figure 1:**
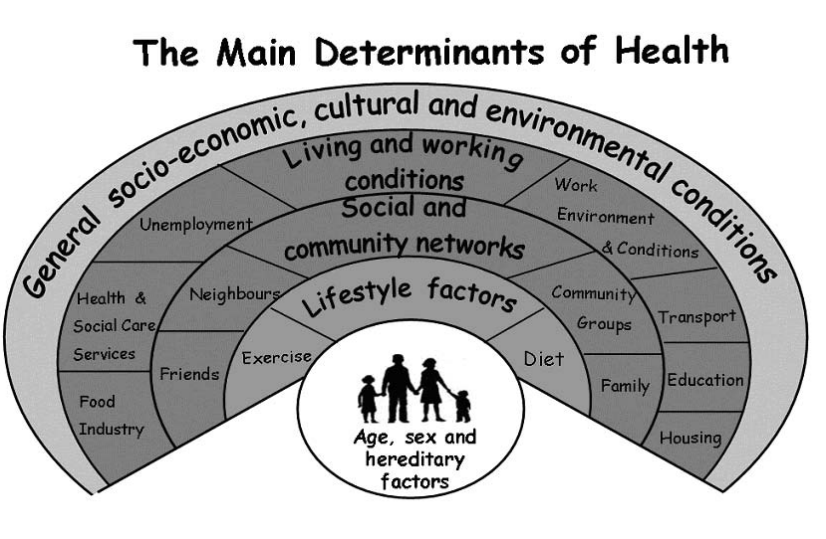
**Picture aid used in qualitative interviews in the pilot study**. Adapted from Dahlgren and Whitehead (with permission) [21].

We were committed to involving users in the design of methods for the KNEPP Study. For part 3 we wanted to undertake qualitative interviews with people about their understanding of the prevention of knee pain and disability in older adults, and what the key determinants of this were. One idea was to use, in the interview setting, a well-established model of health determinants developed Dahlgren and Whitehead [21] (see Figure 1). The model has been applied to theory-based public health policy with specific reference to tackling health inequalities. Although this model is widely adopted within health policy, it has not been applied previously on an individual basis, or in qualitative research. The group discussed whether, or not, it could be used to identify opportunities for prevention of knee pain.

Forum members had wide-ranging experiential knowledge because of their diverse backgrounds and expertise. Consensus soon emerged that the issues identified in Dahlgren and Whitehead's model were relevant to their own specific context. Some participants could associate with the model from their individual perspective but were hesitant about whether it would work as part of a qualitative interview. Key points raised by forum members are outlined below.

- Could the picture be used to evaluate the interview, but not actually during the interview? The researchers would then map the results of the interview to the model as part of the data analysis.

- The picture could be left blank and the respondent asked to colour in what aspects are most important to them.

- Beliefs, attitudes mental well-being were missing from the "rainbow" and there was a need to assess if the person has a positive or negative attitude towards their health.

- The group suggested asking people about how they feel their health and knee pain will be in ten years time and what factors will be important in the future. This cannot be gained from the picture and may add another dimension to the interview and will enable perceptions of prevention of knee pain over a longer period to be investigated.

- Concerns were raised with regard to layout of the model, specific elements of the model that might be not immediately relevant or clear, or the amount of information that the model conveyed.

In addition, the need to provide clear instructions to each layer of the model was emphasised and a small number of changes were agreed upon. For example, addition of the examples of diet and exercise to the lifestyle layer, as this was the only layer with no pre-existing examples.

The forum strongly recommended undertaking pilot interviews. Consensus emerged to test undertaking a traditional open-ended qualitative interview first, and then bring in the model after the patient's initial perspective had been gained. It was also suggested to ask the people who had been interviewed (at the end of the interview), whether, or not, they thought the model was relevant to their experiences.

#### Pilot interviews

In line with the forum's comments and in preparation for part 3 of the KNEPP study, thirteen pilot interviews (8 patients, 5 other stakeholders) were undertaken. Patients were selected from responders to a previous knee pain survey [22] who had given consent to be contacted for further research. The stakeholders were purposively selected to represent key individuals who come into regular contact with people with knee pain. The findings from the pilot interviews were presented to the forum and are illustrated below.

People with knee pain and other stakeholders mentioned many of the key determinants reflected in the model and their impact on health and knee pain prior to the model being introduced in the interview, thus showing the relevance of the model to everyday experiences. Exercise and diet were most often discussed:

"It's difficult within a clinic setting. I stress the importance of exercise. I tend to spell out that certain exercises are more effective than others. Obviously things like weight-bearing exercises, running, etcetera, tend to be not very helpful, and I'll often stress the importance of things like swimming, and demonstrate quadriceps exercises in clinic with the patient as well. I think that's about as far as I go." (Rheumatology Registrar)

Early on in the interviews patients talked about lifestyle factors (for example, diet and exercise) as risk factors for knee pain and targets for prevention:

"I've suffered a little bit and that's because I'd been doing that exercise on – I feel – on treadmills and stepping machines; trying to keep fit and my knee was suffering a bit from it. I've never been that keen on taking tablets. I think if you're overweight, the Doctor is just going to say, "well," you know, "you've got to lose about five stone before it'll be any better." (patient 539)

Occupational factors were also recognised by patients and other stakeholders as illustrated below:

"Well, I don't really know when it started I have had it many years. I used to work on the electricity board and I did a lot of kneeling, fitting electric meters so we spent half of the time kneeling on the floor in the old terrace properties, and I think that is where it initially started from mainly. I retired off the MEB ten years ago, took early retirement, but I worked for the company for 17 years, so for 17 years half of the 17 years was kneeling down, fifty, standing up and kneeling down." (patient 669)

When the model was introduced, many participants elaborated on previous issues raised but were also able to talk about other determinants. In particular, the area of social networks was explored in more detail after the model was introduced, as illustrated below by a patient with knee pain:

"Well, I'm a member (of) a group from the Friendship Club because.. we call them 'wrinklies', you know.. we go to 'wrinklies'...but we have five trips a year and we meet every fortnight; have a cup of tea; bingo or a discussion or sommat, then, this year we're going to Scotland for a.. five days. So that's come into community. Oh I, well, It must contribute. It's.. to some sort, I think, because I mean, I live on my own but very.. very rare stop in. I like be out and about. Oh, I mean, you'd be lost.. I think you'd be lost without 'em.."(patient 2241)

Overall, the interviewees had a positive view of using the model in the interview setting and could relate to it quite easily.

"I think it makes sense, and I will say it makes sense to me because I accrue to the same philosophy and working anyway. So, yes I find this method perfectly acceptable and I can understand, you know, where, these chaps come from because, yes, it's good common-sense. It's very interesting because it, in many ways, echoes what I've just been speaking with you about. I mean, about lifestyle factors; diet and exercise.. yeah.. they agree entirely." (GP)

In addition, all interviewees liked the idea of the model assisting with discussions:

"I think it's useful and I think how we did it where you let.. I think it's always worthwhile gauging what people.. where people are coming from. So, I think I'd probably hold this back in the first instance, let people talk about the impact and what factors they feel are important themselves first. Once that conversation has dried up, to a degree, then perhaps bringing this in and, sort of, on looking upon that, thinking about, you know, "are there any of the factors that they would feel they'd want to talk about" and so forth. But, I think it'ld be interesting to see what they said before and what they said after." (Rheumatology registrar)

Most interviewees felt that the model was comprehensive. One respondent questioned whether "information" was evident in the model but then later felt that this was covered in education. One participant thought that employment should be changed to retirement to make it more relevant to older people. Another respondent felt that mental health issues should be explicit. Although participants were mostly enthusiastic about using the model, there were two areas of uncertainty: the top layer of general socio-economic, cultural and environmental conditions and the food industry section. Only one participant, the Age Concern representative, fully engaged with the top layer without needing explanation and talked about funding priorities and the local Primary Care Trust.

The above results of the pilot interviews were discussed at the 2^nd ^forum meeting and the final decision was to use the model in part 3 of the KNEPP study. The method agreed by members was to start with an open-ended interview and subsequently introduce the model to probe in-depth issues that had been raised, or explore the reasons why certain issues were seen as less important in determining health and prevention of knee pain. A question to ask about health in the next five to ten years was also agreed upon. Uptake of the forum's recommendations regarding methods was explicit and was reflected in the interview schedules. Implementing a constant feedback loop to the forum was critical to demonstrate to the members how their views and recommendations were influencing the research process. This may sound simple but it is actually hard to achieve. Being transparent about the specific contribution of users is vital to guard against tokenism.

### 2. Developing dissemination strategies

Users make a contribution to all aspects of the research process, but particularly to dissemination. Giving advice on the choice and methods used to distribute research findings is a key principle for success in consumer involvement in NHS research [5]. Forum members shared an interest in obesity. Findings from part 1 of the KNEPP study (the systemic review) about obesity as a risk factor for knee pain led to experiential and scientific knowledge being combined to develop a dissemination strategy.

The group felt strongly that research messages should be tailored to different groups. For example, the researchers should develop a clear primary prevention message for those who are not currently overweight or obese, and a specific secondary prevention message about the benefit of weight loss and how this can help with reducing knee pain. In relation to the primary prevention message, scientific knowledge from the systematic review and data modelling in the KNEPP study identified that 19% of new cases of severe knee pain over a 3-year period could potentially be avoided by a one-category shift downwards in people's body mass index. For the purposes of dissemination outside of scientific journals, the forum recommended reporting this as "1 in 5 new cases of severe knee pain..." because this message is clearer to the public. Although many of the participants recognised the health risks of obesity, they were unaware of the extent of its impact on knee pain and disability and wanted to spread this knowledge to their workplace, peer groups and social networks.

There was consensus that stark messages are required using simple and clear wording, and that knee pain is one of many consequences of obesity. Thus, messages about the impact of excess weight on knee problems should be integrated with other health messages. These have to be part of existing information sources, and the group advised the research team to liaise with organisations like Arthritis Research Campaign (**arc**), Diabetes UK, National Obesity Forum and the Department of Health. Targeting a key agency and integrating the knee pain message was seen as a practical way to raise the profile of joint pain and obesity in the general population.

The experiential knowledge of the participants was invaluable when discussing the focus of research messages. The group wanted to stress the benefits of not being overweight, rather than the problems of being overweight, for example, being able to maintain independence, continue gardening, working, be able to get about and climb stairs.

The experiential knowledge gained from the knee pain forum has been disseminated to national organisations like the **arc**. Views have been presented to an expert panel on obesity and OA and have directly influenced research messages for the media and the public on this topic (**arc **booklet on OA and obesity). Few studies on user involvement have involved users in the dissemination of research findings [23] yet we have found particular strengths in this area. Ideas from the group on dissemination are outlined below:

• Share results with different groups (e.g. Arthritis Care).

• Use the media. Hard-hitting messages are required.

• Disseminate using existing outlets e.g. Information section at libraries/post offices etc.

• Education to children and adults, particularly if the focus is primary prevention.

• Use integrated messages. Use other health promotion activities to integrate messages on e.g. benefits of weight loss for diabetes, CHD and knee OA.

• Target sports e.g. kids football. This will help with reducing risk factor like knee injury.

• Targeting occupation related settings:

◦ Workplace initiatives

◦ Work group sessions

◦ Health and safety initiatives

### 3. Identifying research priorities

There are examples in the literature of how patients' demands for research have led to the development of research priorities or new research topics [12,13]. However, there is no good quality evidence on the best method for obtaining views on research priorities (e.g. mailed surveys, telephone discussions or face-to-face meetings) [10]. Caron-Flinterman and colleagues note that user involvement in prioritising is "usually in the form of committees, where it is difficult to determine whether and to what extent the input of patients has influenced decision-making"[12].

The INVOLVE model of user involvement outlines a role for patients in identifying and prioritising research topics [2]. However, training and support for patients is important to facilitate this type of involvement [24]. For example, an understanding of previous research undertaken in the area of interest needs to be obtained, and assistance may be required to help patients translate their views into research questions [24].

In our forum, ideas for future research were gained in two ways. Firstly, discussions were held with the group on their perceptions of prevention of knee pain and risk factors for knee pain. Participants then discussed their own views on what research is now required. This group work was completely user led and the views of the research team on future research were not discussed. The research ideas from the forum are outlined in Table 3.

**Table 3:** Research topics identified by the knee pain forum

**Topic**	**Research ideas**
Changing uncertainty and expectations	- How can we deal with the uncertainty about what can be done for knee pain (patients and professionals)? - How can we overcome the problems of poor expectations in relation to knee pain in society? - How to shift societal expectations?
	
Defining effective interventions	- How to build issues into daily life. This needs studies to make initiatives more appropriate to people - Testing multi-agency working - Long term research required - Why aren't GPs or other health care professionals giving lifestyle advice?
	
Evaluate public health interventions	- Evaluate the results of media and dissemination of research messages. For example, evaluate the outcome of delivering hard hitting messages on behaviour change - Develop community interventions, which need to be free of charge
	
Implementation	- How can we get more evidence into practice?
	
Primary Prevention	- Studies with key groups, like children, younger adults, parents. - What do key groups know about knee pain and the musculoskeletal health? - Do they know of the impact of risk factors for themselves or their children?

Secondly, in one of the pilot study qualitative interviews, a respondent said:

"The NHS seems to be over-burdened with dealing with the people that have the problem. We check machinery, we check cars, we have MOTs and we do all that, but when it comes to ourselves we pay no attention, we wait until we are in trouble" (patient 6028)

This quote led the research team to think about issues of health prioritisation and why patients often de-prioritise their pain [25]. One explanation is that health care organisations and clinicians do not prioritise musculoskeletal pain, [26] and this has impacted on older people's expectations and cultural beliefs about joint pain and other health care conditions.

A proposal was worked up by the researchers into a mixed methods study investigating the extent to which musculoskeletal health features in public health and commissioning decision-making within Primary Care Trusts. The proposal was presented at the final forum meeting to gain further advice about the relevance of this topic. Firstly, participants reinforced the need for a research focus on prevention of joint pain in older adults:

"Prevention's got to be a long-term thing, which, you're not going to see results over the next couple of years are you, it's going to be four or five years down the line, where you might start seeing a drop in, say, knee replacements, so, you've got to spend money now on something that may or may not bring the result later on."

Participants also commented on the research methods proposed as highlighted below:

"I think, you know, a base-line survey of what's going on is a very good idea and I think, you know, case histories are a very good idea, so, who's doing it well and for what reason, what buttons had to be pressed, why did it happen?"

"We don't know in general what PCTs are up to. It might be nice to have the survey to say this is the state of play. What are they doing and what is their focus?"

A further idea was to compare musculoskeletal health with other conditions where National Service Frameworks (NSFs) had already been implemented. For example:

"It'd be interesting to compare it to another chronic disease, you know. I think a lot of these big first NSFs did have a lot of monitoring attached to them, so, you may be able to say well, this is what happened after one NSF."

Through the forum meetings our research ideas were developed using both scientific and experiential knowledge, and because the discussions and decisions were carefully recorded all contributions are easily detectable and transparent.

### Evaluation of engagement

Six members completed the final short evaluation form. It is likely that these members are those who were more engaged with the involvement process generally. However, the responses do give some indication of the impact that involvement had. Overall, for those who returned the form, involvement had been a positive experience. Members felt that their input was valued and had been taken seriously as shown below:

"I have enjoyed the meetings and would be happy to attend other similar projects. Everyone's comments were valued. As I work mostly with 60+ people I get to know about ailments and cures and positive and negative outlooks on life. The discussion also gives me more knowledge which may be helpful in my work." (E6)

This view was reinforced by other members who wrote:

"Overall very good, initially didn't think our views were being taken notice of, but towards the end felt that we were as important as the professional people."(E3)

Some members talked about feeling empowered by being involved in the forum, for example,

"I liked being involved in a project which may in the future influence the policy of health services for people with different forms of knee pain." (E5)

Some members fedback on the social conditions within which the forum was undertaken, for example "a relaxed atmosphere" (E4), "interesting and well prepared and focussed questions" (E6), "good cross section of people in the group" (E3) "liked the exchange of ideas and information" (E2). Importantly some members felt that there should have been more time for discussion (E4, E5, E6). The researchers had planned two-hour sessions (with reading material in between) so as not to overburden members. Those who gave feedback suggested longer meetings in future.

We used the researchers' skills to present the results of ongoing research and to provide interim analysis of the pilot study findings. These elements of our research were not user initiated or controlled as the researchers used their technical knowledge to undertake the systematic review and outlined issues from the literature to facilitate discussions. Overall, however, our involvement strategy has enabled consultation (asking for views and using these to inform decision making), collaboration (active and ongoing partnerships) and a degree of user-controlled research (e.g. through design of research methods).

## Conclusion

We have established a community knee pain forum to work in partnership with researchers on key aspects of knee pain prevention research. Not all parts of the involvement cycle are equally 'weighted' as this depends on the topic, design and methods (level of technical skill required), and preferences of participants. From our experience, people are most interested in agenda setting, shaping specific research tools, commenting on emerging findings, and dissemination. Members appeared content to leave the more technical aspects of research, especially analysis and writing, to the research team. Yet, if material was well prepared and distributed prior to the meeting most forum members were able to offer a critique and substantially contribute to the discussion and formulating conclusions.

Whichever aspect of the cycle is chosen, community engagement requires a range of perspectives (e.g. from users/lay people, clinicians, interest groups and statutory organisations) and the social context requires fostering equal relationships, trust and respect. It is important, therefore, to clearly define the role of facilitators who have to manage the conduct and content of the engagement processes. Careful preparation, sensitivity to individual needs, tailoring support and maintaining contact in between meetings are key parts of this role. Furthermore, engaging with non-academic partners enables mutual learning. Members understand the conduct of research and the limits of funding priorities, and researchers learn about relevance of topics and the importance of outcomes that are meaningful to the range of users. This mutual learning enables enhanced quality of NHS research. Finally, attention needs to be paid as to how user involvement can be sustained beyond one-off projects. In this case, sustainability is ensured in a number of ways: first, the forum will be widened to steer our long-term osteoarthritis research programme; second, two lay members have joined the Centre's Research Users' group that oversees all research programmes and projects; third, the KNEPP project is followed up with a newly funded project on self-care strategies for knee pain and disability and some forum members will serve on its Steering Group. If the accumulated knowledge and experience of users is to be harnessed strategies for long-term involvement are needed before the end of a research project.

## Competing interests

The authors declare that they have no competing interests.

## Authors' contributions

CJ and BNO obtained funding for the KNEPP study and conceived the design of the study. CJ facilitated the forum meetings and participated in drafting the manuscript and interpretation of findings. BNO participated in drafting the manuscript and in interpretation of findings. TO facilitated the forum meetings, undertook the pilot study interviews, participated in drafting the manuscript and in interpretation of findings. All authors have read and approved the final manuscript.

## Pre-publication history

The pre-publication history for this paper can be accessed here:


